# Development of the Dutch Structure for Integrated Children’s Palliative Care

**DOI:** 10.3390/children8090741

**Published:** 2021-08-27

**Authors:** Stephanie Vallianatos, Carolien S. M. Huizinga, Meggi A. Schuiling-Otten, Antoinette Y. N. Schouten-van Meeteren, Leontien C. M. Kremer, A. A. Eduard Verhagen

**Affiliations:** 1 Dutch Knowledge Centre for Children’s Palliative Care, Mercatorlaan 1200, 3528 BL Utrecht, The Netherlands; c.huizinga@kinderpalliatief.nl (C.S.M.H.); m.schuiling@kinderpalliatief.nl (M.A.S.-O.); 2 Princess Maxima Centre for Pediatric Oncology, Heidelberglaaan 25, 3584 CS Utrecht, The Netherlands; A.Y.N.Schouten@prinsesmaximacentrum.nl (A.Y.N.S.-v.M.); l.c.m.kremer@prinsesmaximacentrum.nl (L.C.M.K.); 3 Beatrix Children’s Hospital, University Hospital Groningen, Hanzeplein 1, 9713 GZ Groningen, The Netherlands; a.a.e.verhagen@umcg.nl

**Keywords:** integrated care, children’s palliative care, paediatric palliative care team, homecare, family, care network, life-limiting, life-threatening

## Abstract

Children’s palliative care (CPC) is gaining attention worldwide, facilitated by the exchange of knowledge during regular specialised congresses. This article describes the developments in the Netherlands over the past 15 years. The Foundation for Children’s Palliative Expertise (PAL) was established as a nationwide initiative committed to improving palliative care for children countrywide. This led to the development of the first hospital-based CPC team in 2012, which expanded to a total of seven teams adjacent to children’s university hospitals. Regional networks for CPC were developed in parallel to these teams from 2014 onwards. The networks are a collaboration of professionals from different disciplines and organisations, from hospital to homecare, and have covered the aspects of CPC nationally from 2019 onwards. They are connected through the Dutch Knowledge Centre for CPC. This centre was established in 2018 by the PAL Foundation in collaboration with the Dutch Association for Pediatrics. In 2013, the first evidence-based guideline, ‘palliative care for children’, provided access to knowledge for parents and healthcare providers, and in 2017, a format for an individual palliative care plan was established. Within the Knowledge Centre for CPC, a physician’s support centre for dilemma’s regarding the end of life of children was set up. The efforts to have children’s palliative care embedded in the regular Dutch health care insurance are ongoing.

## 1. Introduction

Before 2007, the care for children with a life-limiting or life-threatening condition in the Netherlands was similar to that in surrounding countries: not a specialty in itself but offered similar to adult palliative care [1]. The palliative care for children was characterised by fragmentation and was not easily available or accessible. Professionals struggled in recognising patients’ care needs beyond medical aspects and often lacked the skills to provide the specific required care. Families of children that were in the palliative phase felt that they were receiving insufficient support during this stressful, uncertain and vulnerable situation [2]. In this article, we will outline the background and developments of CPC in the Netherlands from 2007 until now. The improvement in CPC in the Netherlands started with the best practices in paediatric oncology. This discipline was already more sensitive to the fact that children with uncurable diseases needed palliative care aimed specifically at children and families, with a skilled multidisciplinary approach [3].

Life-limiting or life-threatening conditions are grouped in four categories as used by the Association for Children’s Palliative Care (ACT): “1. Life-threatening conditions for which curative treatment may be feasible but can fail—for example: cancer, organ failure of the heart, liver, kidney; 2. Conditions where premature death is inevitable—children in this category may be severely disabled but have long periods of relatively good health—for example, Duchenne muscular dystrophy and SMA type 1; 3. Progressive conditions without curative treatment options—treatment focused on palliative care may last for many years—for example, severe metabolic conditions; 4. Irreversible but non-progressive conditions causing severe disability, leading to susceptibility to health complications and likelihood of premature death—for example, complex disabilities such as brain or spinal cord injury” [4].

### 1.1. Children’s Palliative Care Worldwide

CPC is an integrated approach for children with a life-limiting or life-threatening condition, focusing on quality of life and good circumstances around death. Globally, we embrace the following World Health Organisation definition of CPC [1]: “Palliative care for children is the active total care of the child’s body, mind and spirit, and also involves giving support to the family. It begins when illness is diagnosed, and continues regardless of whether or not a child receives treatment directed at the disease. Health providers must evaluate and alleviate a child’s physical, psychological, and social distress. Effective palliative care requires a broad multidisciplinary approach that includes the family and makes use of available community resources; it can be successfully implemented even if resources are limited. It can be provided in tertiary care facilities, in community health centres and even in children’s homes.” Worldwide initiatives have evolved from the urgent need for progress in the field of CPC with more information, formalised networks and research to achieve the best possible quality of life for children with life-threatening conditions and for their families [4,5].

**Figure 1:**
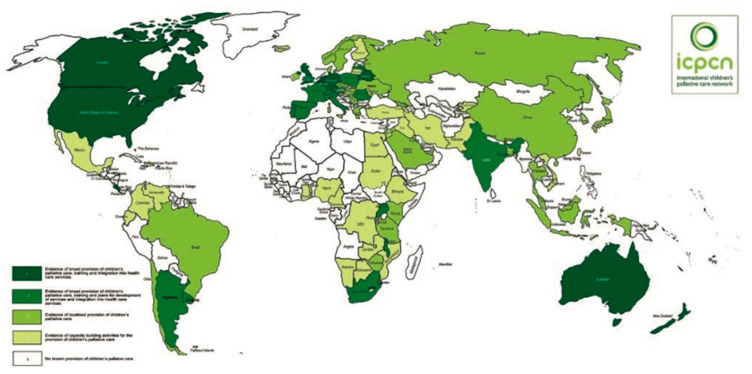
Situation in 2019 of provision of CPC around the world, reprinted with permission from ref. [6]. Copyright 2019 ICPNC. The dark green colour indicates broad availability of CPC in several places, but there is still room for improvement.

CPC worldwide is a young discipline. Apart from England, specific provision of CPC was hardly visible in 1980 [6]. The first children’s hospice, the Helen House, opened in Oxfordshire (UK) in 1982. The International Children’s Palliative Care Network (ICPCN) was founded in 2005. The goal of the ICPCN is: “to achieve the best quality of life and care for children and young people with life-limiting conditions, their families and carers worldwide. Important tasks are improving awareness, lobbying for the global development of CPC services and sharing expertise, skills and knowledge”. [7]. Meanwhile the provision of CPC has improved worldwide, as demonstrated by the geographical map depicting 2019 in Figure 1.

### 1.2. Situation CPC in the Netherlands

In the Netherlands, 5000–7000 children and their families are eligible for palliative care; and approximately 23% of these children have an oncological disease, and 77% have a neonatal, neurological or metabolic disease, often in chronic condition. Annually, in total, around 1100 children (0–20 year) die [8]. Subsequently more chronic conditions and rare diseases in children are emerging the coming decades [9,10]. About 10.000 children with chronic conditions are known in hospitals and home care who are cared for and nursed over a period of many years. About 65% of this group is palliative, based on the WHO definition of paediatric palliative care [1]. In comparison, we highlight some figures about palliative care for adults: 110,000 adults per year receive palliative care in the Netherlands, most often because of an oncological disease, cardiac or vascular disease, respiratory disease and/or dementia [11]. Furthermore, there is a wide range of knowledge and education available about the specific palliative care for adults.

For a long time, palliative care for adults was applied more or less identically to children, and specialised health care providers focusing on CPC were lacking. However, specialised care is needed because children are not young adults. CPC differs from palliative care for adults for several reasons: children in need of palliative care often suffer from different diseases compared to adults, and these diseases are rare, complex, chronic and progressive, with many different symptoms [9]. The care for these children is complex (‘high tech, high care’), and their leading care pediatrician is often affiliated with a university hospital. Knowledge about how to provide the best CPC was limited to the group of caregivers who provided it at the time before 2007. Knowledge was not exchanged, and the necessary knowledge in all domains for caregivers was not accessible. Moreover, if caregivers had any knowledge, it was based only on existing experience, not scientific research [2]. In addition, the care for children, in general, requires special expertise because ill children are part of a family system. There are multiple dynamics between the child, parents and possible siblings. Even if a child is confronted with illness and limitations, they will continue to develop at the age-appropriate physical, emotional, cognitive and social levels [12,13]. Research has shown that parents often feel pressure to take on a coordinating role, a complex and intensive task [14,15]. A central point of contact appears to be one of the most important needs of parents, but this need could rarely be met. Other needs, such as the care and guidance of other siblings or aftercare, were also hardly met [7].

### 1.3. Exploration of an Appropriate Approach of CPC

In 2007, the Foundation for Children’s Palliative Expertise (PAL) was established. PAL is a nationwide foundation committed to improving palliative care for children in the Netherland, with the aim of offering children with a life-threatening or life-limiting condition the highest possible quality of life. The care for a child in need of palliative care, in or outside their home, requires a coordinated approach to support the whole family. Due to the activities of the PAL Foundation, considerable improvements in homecare have been realised—for example, building expertise by initiating networks and organising training. In the early days of CPC, several initiatives were implemented within the field of paediatric oncology to improve the coordination and organisation of CPC. For example, multidisciplinary case debriefings were held after the death of a child. From these ‘end-of-life- care debriefings’, suboptimal care coordination was found to be an important cause of communication problems among professional caregivers. Other authors have confirmed that poor coordination of care is a major cause of inadequate CPC [16].

A guideline for paediatric oncology was developed with points of interest for the different domains of palliative care. This guideline was the basis for an individualised paediatric palliative care plan per patient that was later developed for the implementation of CPC in daily practice [17]. Increasing numbers of options and facilities became available to treat children at home as much as possible and allow them to remain with their families. Within our Dutch network, there is nationwide specific homecare with specialised paediatric homecare nurses, a wide range of schools for children with special needs and nursing child day-care facilities, and there are 12 children’s hospices and 1 hospice for young adults (16 to 35 y).

The lack of knowledge and lack of coordination led to the need to improve CPC with a focus on making knowledge centrally available and easily accessible and on setting up CPC teams and CPC networks in seven regions. The result was a nationwide structure of integrated CPC, as described in the following chapters. In 2018, the PAL Foundation, in collaboration with the Dutch Association for Pediatrics, became the Dutch Knowledge Centre for Children’s Palliative Care.

## 2. Approach

In this chapter, we describe the creation of integrated CPC in the Netherlands, which started in 2012. To achieve our aim, from scratch, a nationwide structure for integrated CPC with substantive quality, coordination and continuity was built. First, we started to analyse what was needed and what we could learn from good practices. Moreover, we started to increase awareness through communication and education. In 2012, the first CPC team in the Emma Children’s Hospital in Amsterdam started. On the basis of this project, a model was developed with the provision of expert CPC, regardless of where the child is staying, at home, hospice or hospital [18]. We found that care close to home was very fragmented and that it was difficult to connect professionals with other domains of normal life, such as school, work and sports. The CPC team facilitates the bridge from hospital to home. The development of this first CPC team was extensively evaluated and researched. Research showed that this specialised CPC team has a positive effect on the satisfaction regarding care, symptom management and quality of life and thus also leads to fewer hospital admissions and a cost reduction [19,20,21,22]. The results of this research have directed the development of the other CPC teams.

Parallel to this project, the first evidence-based guideline for CPC was developed. In 2013, the Dutch Association of Paediatrics finalised the guideline ‘Palliative care for children’ [23]. This open-access document was produced in collaboration with all parties involved in CPC and intended to improve the quality of CPC and the effectiveness of collaboration. This guideline is more or less a quality framework: in addition to recommendations for medical care, it also contains recommendations for the organisation of CPC—for example, organising CPC close to home as much as possible. Therefore, the next step was to develop cross-domain, regional networks of professionals involved in the care and guidance of families with a seriously ill child at home. The general practitioner and paediatric homecare nurses have an important and coordinating role in this setting, and caregivers from different disciplines are available for children and parents depending on their care needs. In 2014, a pilot project of such a network started in the region around the Children’s University Hospital in Leiden. The development and evaluation of the regional CPC networks are based on the Development model for integrated care [24]. From this model, 10 steps towards a CPC network have been elaborated [25].


  CPC teams in hospitals: This is a multidisciplinary team consisting of specialised children’s nurses, paediatricians, psychologists and child life specialists and is the bridge between hospital and home. The team offers support and guidance to families and first-line care by the family physician and homecare team. This care demands a professional and coordinating approach to support the whole family during the difficult process of palliative care.
  CPC networks: This is a collaboration of professionals from different disciplines (specialised nurses, paediatric homecare nurses, paediatricians, general practitioners, social workers, psychologists, paramedics, child life specialists, day care facilities,
bereavement care, spiritual workers, client support) and organisations involved in care from hospital to home, with specific expertise in caring for families with a severely ill child. These cross-domain networks offer support and guidance for families and focus on balance and everyday life for the whole family. Moreover, the networks fulfil a free access consultation function for parents and professionals who have questions about CPC. Another important task of the CPC networks is to increase and exchange expertise in the region via regular interprofessional meetings.

**Figure 2:**
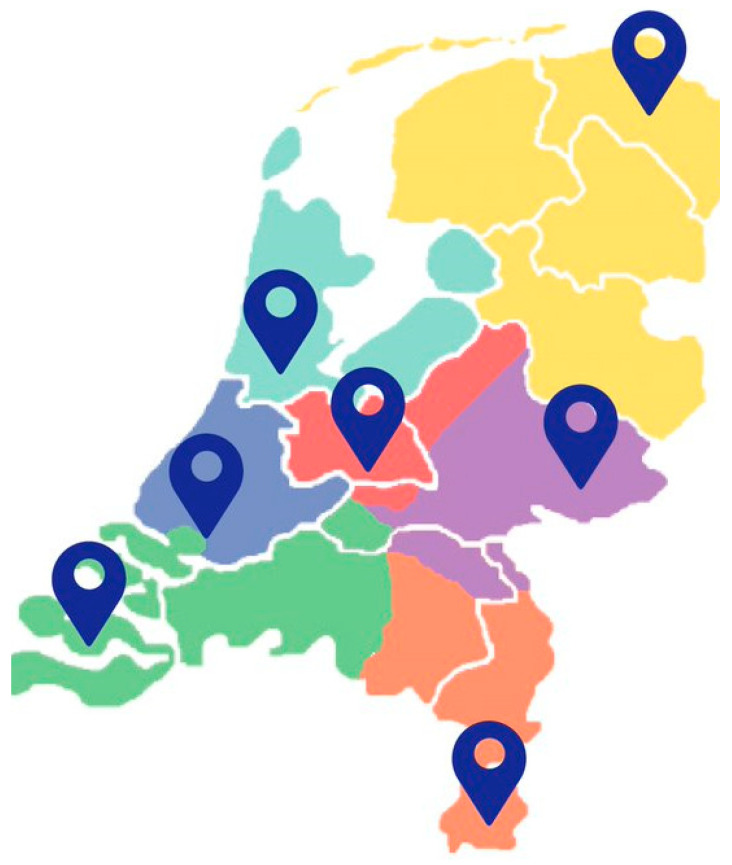
Map of the Netherlands with the seven areas of the nationwide structure CPC.

These pilots led to the development of a nationwide structure. The structure is organised in seven regions around the seven University Children’s Hospitals. In Figure 2, we present a map of the Netherlands, highlighting the seven areas. Between the CPC teams and CPC networks in the area, cross-fertilisation is rapidly taking place.

Another important development concerns the development of an individualised paediatric palliative care plan format with extra attention given to Advance Care Planning (ACP) [16]. The individual care plan is seen as the best tool to translate the general recommendations from the guideline to specific policy at the bedside. In this plan are both the child and family’s needs and desires and the recommendations of the evidence-based guideline ‘palliative care for children’ included. In practice, the individual care plan is discussed with the child and parents actively around the transfer from hospital to home, and can be changed according to mutual insights, after which the care plan is approved. The voices of the child and their parents are heard in the preparation of this plan. Active discussion about new or altered health problems of the child and new requirements in care can be brought up by anyone involved.

### Evaluation and Improvement

The development of the CPC teams and CPC networks is ongoing. The knowledge gained from the experiences of parents is an important input for improvements. To this end, we regularly organise mirror meetings. Mirror meetings are guided conversations with a two-circle set up to shed light on how clients experience the received support [26]. In the inner circle, conversations with the parents take place. An independent moderator leads the conversation about how parents (have) experience the care and support and any requests or advice they have. In the outer circle, the professionals of the CPC teams and CPC networks are present as listeners. This is with the aim of making the professionals aware of the perspectives of the parents in a safe environment. This direct feedback provides a very stimulating effect for improvement. A work session is held immediately after the mirror meeting. During this, the professionals and parents work together to formulate improvement actions for specific topics.

## 3. Results

In the Netherlands, simultaneously with the development of CPC-teams by the children’s university hospitals, professionals in hospitals and homecare have developed networks of integrated CPC (first in 2014). The aim was to realise the coordination and continuity of care close to home and to increase expertise. In 2020, the nationwide structure with seven networks and seven CPC-teams was completely realised. The Knowledge Centre for CPC with the CPC structure in the regions provides expertise, education, consultation, networking, guidelines, policy development, research and innovation.

The established values since the development of CPC in the Netherlands are:

- Recognition of CPC as a specialised field of care;
- Focus not only on medical aspects but an integrated multidisciplinary approach;
- Focus on the whole family: the ill child, parents and siblings;
- Care can be given at home more often; fewer (re)admissions to hospital;
- A careful transfer of a palliative process from hospital to home, actively prepared and discussed with child and parents;
- Space for daily family life by relieving of parents from organisational tasks and arranging care at home;
- Provision of bereavement care and aftercare.

**Figure 3:**
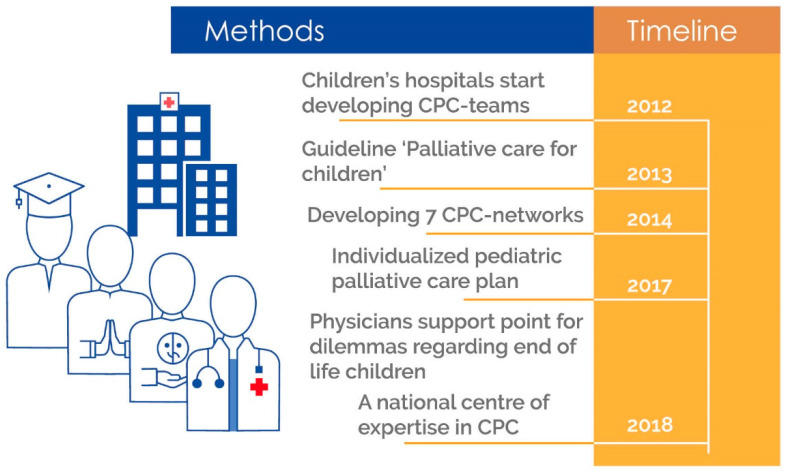
Timeline creation of the nationwide structure of CPC in the Netherlands.

Moreover, with the recognition of CPC, attention has been paid to ethical dilemmas, for which an easily accessible consultation point has been set up for professionals for dilemmas regarding end-of-life questions for children. Furthermore, CPC is embedded and funded by insurance companies. The Knowledge Centre for CPC and the CPC networks are funded by the government. For the CPC teams in the university hospitals, the realisation of sustainable financing is underway. Figure 3 shows the timeline of the results for improving CPC in the Netherlands.

### Recent Developments

The original target group for the CPC teams was children with a life-limiting or life-threatening condition. Last year, the CPC networks expanded their target group to children who are chronically and seriously ill but whose condition is not life-threatening or life-limiting. These children and their families often receive complex and long-term multidisciplinary care at home and experience similar problems regarding arranging and coordinating care as families in CPC. Moreover, the healthcare organisations that provide the care are already active members collaborating in the CPC networks. The nationwide structure creates greater transparency and collaboration. For both children and their families, it has resulted in a better balance and greater focus on daily family life. Furthermore, the guideline ‘palliative care for children’ and the individual palliative care plan are currently being further improved in detail, based on research and the experiences of parents and professionals.

## 4. Conclusions

We conclude that progress has been made in improving and integrating CPC in the Netherlands. CPC is now recognised as a specialised field of care. Seven hospital-based CPC teams and seven regional CPC networks in our country coordinate and provide the care in close collaboration with parents, paediatric homecare nurses, general practitioners and other professional caregivers. Knowledge is centralised and easily accessible for both parents and caregivers. To ensure that the structure continues and further develops, the Dutch Knowledge Centre for CPC had been created and will provide a basis to continue and improve the CPC-structure and expertise. An important success factor for realising this nationwide structure of CPC teams and networks and the knowledge centre has been the development of the guideline ‘palliative care for children’ in 2013. It has formed the basis for improving the quality, organisation and financing of CPC.

Dutch CPC has developed towards the WHO definition and ambition, and it now encompasses much more than terminal care. With the nationwide structure, we reach a broad group of children with a life-limiting or life-threatening condition and also seriously ill children which chronic illnesses who experience similar problems. Before, this was inconceivable. A nationwide structure and regional collaboration offer a strong basis for the well-organised guidance and support of children in need of CPC and their families and caregivers.
